# NOD/SCID-GAMMA Mice Are an Ideal Strain to Assess the Efficacy of Therapeutic Agents Used in the Treatment of Myeloma Bone Disease

**DOI:** 10.1371/journal.pone.0119546

**Published:** 2015-03-13

**Authors:** Michelle A. Lawson, Julia M. Paton-Hough, Holly R. Evans, Rebecca E. Walker, William Harris, Dharshi Ratnabalan, John A. Snowden, Andrew D. Chantry

**Affiliations:** 1 Department of Oncology, University of Sheffield, Sheffield, United Kingdom; 2 Mellanby Centre for Bone Research, University of Sheffield, Sheffield, United Kingdom; 3 Department of Haematology, Sheffield Teaching Hospitals NHS Foundation Trust, Royal Hallamshire Hospital, Sheffield, United Kingdom; Jilin University, CHINA

## Abstract

Animal models of multiple myeloma vary in terms of consistency of onset, degree of tumour burden and degree of myeloma bone disease. Here we describe five pre-clinical models of myeloma in NOD/SCID-GAMMA mice to specifically study the effects of therapeutic agents on myeloma bone disease. Groups of 7–8 week old female irradiated NOD/SCID-GAMMA mice were injected intravenously via the tail vein with either 1x10^6^ JJN3, U266, XG-1 or OPM-2 human myeloma cell lines or patient-derived myeloma cells. At the first signs of morbidity in each tumour group all animals were sacrificed. Tumour load was measured by histological analysis, and bone disease was assessed by micro-CT and standard histomorphometric methods. Mice injected with JJN3, U266 or OPM-2 cells showed high tumour bone marrow infiltration of the long bones with low variability, resulting in osteolytic lesions. In contrast, mice injected with XG-1 or patient-derived myeloma cells showed lower tumour bone marrow infiltration and less bone disease with high variability. Injection of JJN3 cells into NOD/SCID-GAMMA mice resulted in an aggressive, short-term model of myeloma with mice exhibiting signs of morbidity 3 weeks later. Treating these mice with zoledronic acid at the time of tumour cell injection or once tumour was established prevented JJN3-induced bone disease but did not reduce tumour burden, whereas, carfilzomib treatment given once tumour was established significantly reduced tumour burden. Injection of U266, XG-1, OPM-2 and patient-derived myeloma cells resulted in less aggressive longer-term models of myeloma with mice exhibiting signs of morbidity 8 weeks later. Treating U266-induced disease with zoledronic acid prevented the formation of osteolytic lesions and trabecular bone loss as well as reducing tumour burden whereas, carfilzomib treatment only reduced tumour burden. In summary, JJN3, U266 or OPM-2 cells injected into NOD/SCID-GAMMA mice provide robust models to study anti-myeloma therapies, particularly those targeting myeloma bone disease.

## Introduction

Multiple myeloma (MM) is a cancer of differentiated B-lymphocytes leading to the clonal expansion of plasma cells in the bone marrow (BM). Despite continually improving treatments, myeloma is almost always incurable. One of the most debilitating features of MM is the development of osteolytic bone disease, which results in increased susceptibility to bone fractures, bone pain and hypercalcaemia. A number of pre-clinical animal models of MM have been developed to assess the efficacy of therapeutic agents used in the treatment of myeloma bone disease (MBD) [1–7]. Most recently the immune-suppressed NOD/SCID-GAMMA (NSG) strain of mice has been used successfully in human xenograft models of MM. In these studies, a number of myeloma cell lines [8–12] and patient-derived myeloma cells [10–12] were injected into NSG mice leading to varying levels of BM infiltration. The effect of anti-tumour agents on the growth of myeloma cells and the overall survival of animals has also been assessed in various NSG models [8, 9, 13–15]. However, there is limited information on the development of osteolytic disease in these models [3, 11, 16, 17]. Further investigation is required to identify and validate the best models in terms of consistency of onset, degree of tumour infiltration and extent of MBD.

In 2004, Miyakawa *et al*. [16] were the first group to inject U266 cells (a myeloma cell line) into NSG mice via the tail vein. This showed U266 infiltration of the BM at the end stages of disease by fluorescence-activated cell sorting (FACS) using an anti-human CD45 antibody and by the presence of human IgE (which is produced by the U266 cells) in histological BM sections. They also demonstrated, by histological analysis, that U266 BM infiltration caused the development of osteolytic lesions. Similarly Hofgaard *et al*. [3] showed that BM infiltration of MOPC315.BM.Luc cells (a mineral oil-induced plasmacytoma) in NSG mice resulted in an increase of tartrate-resistant acid phosphatase (TRAP) positive osteoclasts in histological sections. However, assessment of bone disease in both of these models was limited to histological analysis only. More recently, Hurchla *et al*. [17] performed micro-computed tomography (micro-CT) analysis to assess bone disease in NSG mice intravenously injected with RPMI-8266 cells, which resulted in BM infiltration and osteolytic disease. Treating these mice with oprozomib, a proteasome inhibitor, reduced the tumour growth of myeloma cells in the BM when assessed by fluorescent imaging and analysis of human IgGλ levels in serum. Furthermore, micro-CT analysis showed that oprozomib prevented tumour-induced trabecular bone loss and reduced serum levels of the bone turnover marker carboxy terminal telopeptide (CTX) as well as increasing levels of pro-collagen 1 N-terminal peptide (P1NP).

Taken together, there is limited information on MBD in NSG mice injected with myeloma cell lines or patient-derived cells. There is therefore a prerogative to identify and validate the best murine myeloma models in terms of consistency of tumour burden and extent of MBD. Consistency and reliability between animals is essential for meaningful pre-clinical evaluation of anti-myeloma therapies, particularly those targeting MBD.

The aim of this report was firstly, to determine the time to onset of disease, the variability in tumour burden and the extent of bone disease in NSG mice injected with 4 different myeloma cell lines or patient-derived cells; and secondly, to assess the use of NSG myeloma models for the evaluation of bone therapeutics. We hypothesised that NSG mice injected with a BM-derived myeloma cell line (JJN3) would develop severe bone disease at a faster rate than mice injected with peripheral blood-derived cells (U266, XG-1, OPM-2 and patient-derived cells). In view of the potential implications for pre-clinical testing of anti-myeloma agents, we also investigated whether administration of two bone-modulating agents currently used in the clinic to treat MM (a bisphosphonate, zoledronic acid, and a proteasome inhibitor, carfilzomib) would prevent MBD in 2 of these models.

## Materials and Methods

### Ethics statement

All procedures involving animals were approved by the Home Office (PPL 40/3462) and the University of Sheffield’s Animal Ethics Committee. Patient cells were acquired with appropriate ethical permission (REC reference: 05/Q2305/96). All participants provided written consent to participate in this study. Original consent forms are stored in a secure location and patient demographics and disease features entered into an encrypted database governed by the Research and Development Service Sheffield Teaching Hospitals, NHS Foundation Trust UK. This consent procedure was approved by the South Sheffield Research Ethics Committee in August 2005 and subsequently ratified by the NHS Health Research Authority, National Research Ethics Committee Yorkshire and the Humber—Sheffield in November 2012.

### Myeloma cell lines and patient-derived cells

JJN3 cells (derived from the BM of a 57-year-old woman with plasma cell leukaemia at diagnosis) and OPM-2 cells (derived from the peripheral blood of a 56-year-old woman with MM in the terminal leukaemia phase) were purchased from DSMZ (Germany). U266 cells (derived from the peripheral blood of a 53-year-old man with MM) were purchased from LGC Standards (UK) and XG-1 cells (an IL-6 dependent human myeloma cell line) were a kind gift from John Shaugnessy, Little Rock, USA. Cell lines were genetically profiled by DSMZ and ATCC using short tandem repeat analysis to confirm their identity. Patient-derived myeloma cells were acquired from BM aspirates of patients, where CD138 positive (CD138^+^) cells were collected using magnetic microbeads (MACS, Miltenyi Biotec). The patient cells used in these studies were from a Caucasian 70-year old male with plasma cell leukaemia.

### Mice

NSG (NOD.Cg-Prkdcscid Il2rgtm1Wjl/SzJ) mice were purchased from Charles River laboratories (UK).

### Study to compare time to onset of disease, tumour burden and bone disease

Groups of 7–8 week old female NSG mice (n = 3–5/group) were administered by intravenous injection via the tail vein with 100 μl phosphate buffered saline (PBS, naïve control), 1x10^6^ JJN3, U266, XG-1, OPM-2 cells or patient-derived myeloma cells. At the first signs of morbidity in each tumour group, all animals were sacrificed with a control group.

### Assessment of bone disease and tumour burden

At sacrifice, the right tibiae were dissected free of soft tissue and fixed in 10% formalin before micro-CT analysis was used to measure the percentage of trabecular bone volume (BV/TV, %), trabecular number (Tb. N, mm^-1^) and the cortical bone volume (C. BV, mm^3^). The number and area of cortical bone lesions (pixels^2^) were assessed by taking the micro-CT datasets, removing the trabecular bone and then rendering the datasets to create 3D models using Drishti (version 1.0, ANUVizlab, Australia), followed by analysis of 3 different sides of the bone using ImageJ (version 1.47, NIH, USA). After decalcification, wax embedding and sectioning of the tibiae, the numbers of osteoclasts (following TRAP staining) and osteoblasts on the cortico-endosteal surfaces were assessed using standard histomorphometric methods [18]. Tumour burden was assessed on sections of tibiae that had been stained with haematoxylin and eosin, where the distinct morphology of the myeloma cells distinguishes them from normal marrow. The proportion of BM occupied by the myeloma cells was assessed using OsteoMeasure Advanced Bone Histomorphometry Video System (Osteometrics, Inc. Decatur, GA, USA) and expressed as a percentage of the whole bone section area.

### A therapeutic study using JJN3-bearing mice treated with zoledronic acid once tumour was established

Female NSG mice (7–8 weeks old) were split into 3 groups (n = 5/group). Group 1 was a non-tumour bearing control group (Naïve). Group 2 was injected via the tail vein with 1x10^6^ JJN3 cells and treated 13 and 15 days later, once tumour was established, with PBS subcutaneously (JJN3). Group 3 was injected via the tail vein with 1x10^6^ JJN3 cells and treated 13 and 15 days later, once tumour was established, with zoledronic acid (Proctor & Gamble, 125 μg/kg subcutaneously twice a week) (JJN3-Zol). At the first signs of morbidity (after 3 weeks) all animals were sacrificed. Bone disease and tumour burden were assessed as described above.

### A therapeutic study using JJN3-bearing mice treated with zoledronic acid at the time of tumour cell injection or carfilzomib once tumour was established

Female NSG mice (7–8 weeks old) were split into 4 groups (n = 8/group). Group 1 was a non-tumour bearing control group (Naïve). Group 2 was injected via the tail vein with 1x10^6^ JJN3 cells and treated with vehicle (JJN3). Group 3 was injected via the tail vein with 1x10^6^ JJN3 cells and from 7 days post tumour cell injection treated for 2 weeks with carfilzomib (Selleckchem, 1 mg/kg in 10% 2-hydroxypropyl-β-cyclodextrin in 0.01 M citrate buffer pH 3.5, intravenously twice a week) (JJN3-Car). Group 4 was injected via the tail vein with 1x10^6^ JJN3 cells and treated from the time of tumour cell injection with zoledronic acid (125 μg/kg subcutaneously twice a week) (JJN3-Zol). At the first signs of morbidity (after 3 weeks) all animals were sacrificed. Bone disease and tumour burden were assessed as described above.

### A therapeutic study using U266-bearing mice treated with zoledronic acid at the time of tumour cell injection or carfilzomib once tumour was established

Female NSG mice (7–8 week old) were split into 4 groups (n = 8/group). Group 1 was a non-tumour bearing vehicle control (Naïve). Group 2 was injected via the tail vein with 1x10^6^ U266 cells and treated with vehicle (U266). Group 3 was injected via the tail vein with 1x10^6^ U266 cells and from 6 weeks post tumour cell injection treated for 2 weeks with carfilzomib (Selleckchem, 3 mg/kg in 10% 2-hydroxypropyl-β-cyclodextrin in 0.01 M citrate buffer pH 3.5, intravenously twice a week) (U266+Car). Group 4 was injected via the tail vein with 1x10^6^ U266 cells and treated from the time of tumour cell injection with zoledronic acid (125 μg/kg subcutaneously twice a week) (U266+Zol). At the first signs of morbidity all animals were sacrificed. Bone disease and tumour burden were assessed as described above.

### Statistical analysis

Data were analysed using either a Mann-Whitney test or a Kruskal-Wallis test with a Dunn’s multiple comparisons test calculated using GraphPad Instat version 6.0b (California, USA). All data are expressed with error bars representing standard error of mean (SEM).

## Results

We have determined the time to onset of disease, the variability in tumour burden and the extent of bone disease in NSG mice injected with 4 different myeloma cell lines and patient-derived cells compared to non-tumour control mice.

### Injection of JJN3, U266 or OPM-2 cells into NSG mice results in high tumour burden and severe bone disease with low variability in tumour burden, whereas injection of XG-1 and patient-derived cells results in lower bone marrow infiltration and bone disease with higher variability

**Fig 1:**
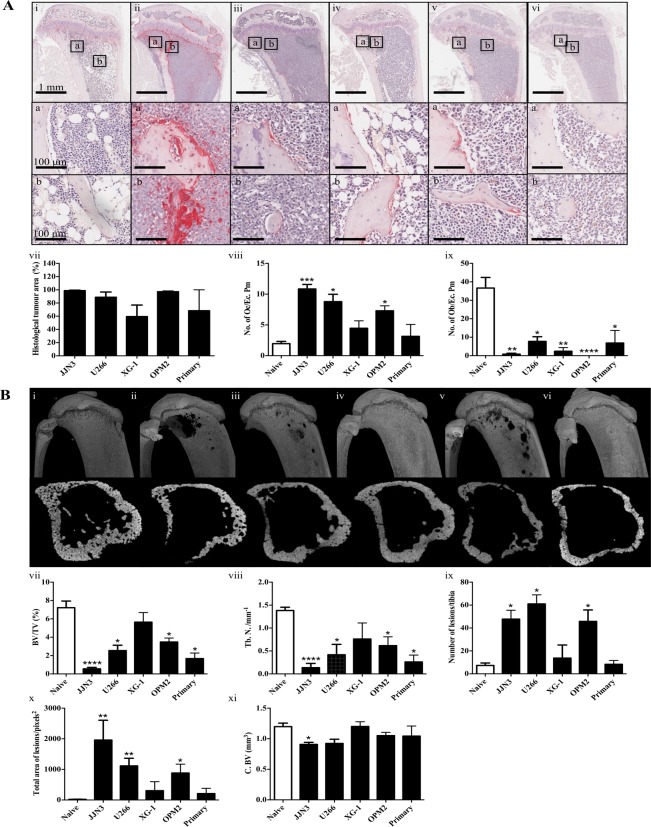
Injection of JJN3, U266, XG-1, OPM-2 cell lines and patient-derived myeloma cells into NSG mice results in varying levels of bone marrow infiltration and osteolytic disease. **A**. Representative histology images of longitudinal images and insets of tibiae from NSG mice injected with 100 μl PBS (Naïve) (i), 1x10^6^ JJN3 (ii), U266 (iii), XG-1 (iv), OPM-2 (v) or patient-derived myeloma cells (vi) at the end stages of disease. Insets below show the endo-cortical (box a) and trabecular (box b) bone regions. The percentage histological tumour area (vii), the number of osteoclasts (No. of Oc./Ec. Pm) (viii) and osteoblasts (No. of Ob./Ec. Pm) (ix) on the cortico-endosteal surface. **B**. Representative longitudinal and cross-sectional micro-CT images of tibiae from NSG mice injected with 100 μl PBS (Naïve) (i), 1x10^6^ JJN3 (ii), U266 (iii), XG-1 (iv), OPM-2 (v) or patient-derived myeloma cells (vi) at the end stages of disease. Micro-CT analysis of trabecular bone volume (BV/TV, %) (vii), trabecular number (Tb. N., mm^-1^) (viii), the number of cortical bone lesions (ix), the total lesion area (pixels^2^) (x) and the cortical bone volume (C. BV, mm^3^) (xi). Data are presented as mean±SEM and significance from the non-tumour control group (Naïve) is indicated, where *p<0.05, **p<0.01, ***p<0.001 and ****p<0.0001.

Injection of JJN3 cells into NSG mice resulted in an aggressive, short-term model of myeloma with mice exhibiting signs of morbidity 3 weeks after tumour cell injection. In contrast, injection of U266, XG-1, OPM-2 or patient-derived myeloma cells resulted in less aggressive longer-term models with mice exhibiting signs of morbidity 8 weeks after tumour cell injection. In addition, we have injected other primary patient-derived cells (from patients with plasma cell leukaemia or relapsed MM) into NSG mice and despite all cells infiltrating the BM and causing MBD the variability for both was high (results not shown), similar to the patient sample shown in Fig. 1. Tumour infiltration of the BM can be seen in longitudinal sections of tibiae from all 5 models compared to non-tumour control mice (Fig. 1Ai-vi). Tumour burden in these bone sections varied between 98.72±0.78% and 59.45±17.50% in JJN3 or XG-1 injected mice, respectively (Fig. 1Avii). Mice injected with JJN3, U266 and OPM-2 cells showed the lowest variability in tumour burden, whereas the XG-1 and patient-derived cells showed the most. A similar variation in tumour burden was also seen by FACS analysis using a human specific antibody (results not shown). Tumour infiltration of the BM was also observed in the skull and lumbar vertebrae (results not shown).

Histological analysis also showed that tumour infiltration caused a significant increase in osteoclast numbers (Fig. 1Aviii) in mice injected with JJN3, U266 and OPM-2 cells, with no significant increase observed in XG-1 or patient-derived infiltrated BM sections. Interestingly, tumour infiltration of the BM also caused a significant decrease in osteoblast numbers on the cortico-endosteal bone surface in all the myeloma models (Fig. 1Aix).

The extent of the bone disease in each model was assessed by micro-CT (Fig. 1Bi-vi). A significant reduction in trabecular bone (Fig. 1Bvii) and in trabecular number (Fig. 1Bviii) was observed in all myeloma treated animals except for those injected with XG-1 cells.

Cortico-endosteal measurements showed tibiae infiltrated with JJN3, U266 or OPM-2 cells had significant numbers of osteolytic lesions compared to non-tumour controls (Fig. 1Bix & x), whereas tibiae infiltrated with XG-1 or patient-derived myeloma cells showed no significant bone lesions compared to controls. Despite this, analysis of the cortical bone volume only showed a significant reduction in JJN3-infiltrated tibiae compared to the non-tumour controls (Fig. 1Bxi).

Taken together, these results show the optimal models to use to study MBD are NSG mice injected with JJN3 cells for a short-term model, and U266 or OPM-2 cells for long-term models, given the low variability demonstrated in both tumour burden and MBD.

### Zoledronic acid prevents JJN3-induced bone disease but does not reduce tumour burden, whereas carfilzomib reduces tumour burden but does not significantly reduce bone disease

**Fig 2:**
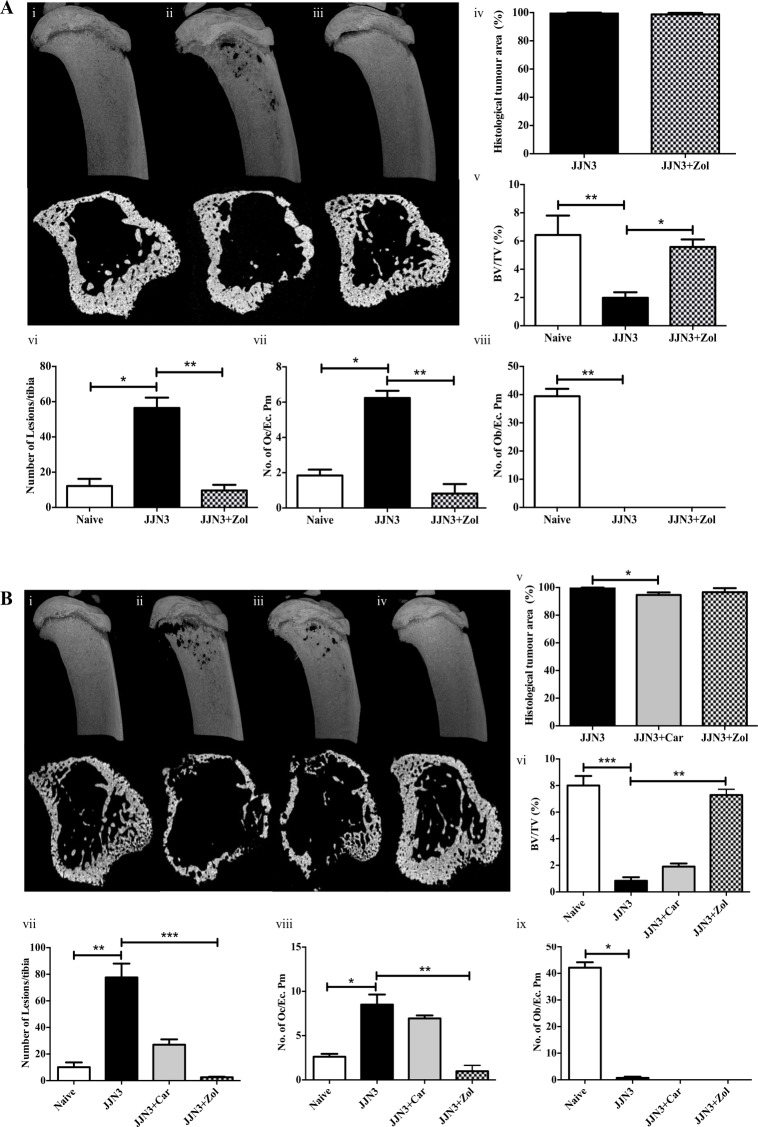
Zoledronic acid prevents JJN3-induced bone disease and carfilzomib reduces tumour burden. **A**. Representative longitudinal and cross-sectional micro-CT images of tibiae from NSG mice at the end stage of disease (3 weeks post-injection of tumour cells), injected with vehicle (Naïve) (i), 1x10^6^ JJN3 cells (JJN3) (ii) or JJN3 cells and treated with zoledronic acid once tumour was established (JJN3+Zol) (iii). The percentage histological tumour area (iv), micro-CT analysis of trabecular bone volume (BV/TV, %) (v) and the number of cortical bone lesions (vi), and the number of osteoclasts (No. of Oc./Ec. Pm) (vii) and osteoblasts (No. of Ob./Ec. Pm) (viii) on the cortico-endosteal surface. **B**. Representative longitudinal and cross-sectional micro-CT images of tibiae from NSG mice at the end stage of disease, injected with vehicle (Naïve) (i), 1x10^6^ JJN3 cells (JJN3) (ii), JJN3 cells and treated with carfilzomib once tumour was established (JJN3+Car) (iii) or JJN3 cells and treated with zoledronic acid from the time of tumour cell injection (JJN3+Zol) (iv). The percentage histological tumour area (v), micro-CT analysis of trabecular bone volume (BV/TV, %) (vi) and the number of cortical bone lesions (vii), and the number of osteoclasts (No. of Oc./Ec. Pm) (viii) and osteoblasts (No. of Ob./Ec. Pm) (ix) on the cortico-endosteal surface. Data are presented as mean±SEM and a significant difference from the tumour control group (JJN3) is indicated, where *p<0.05, **p<0.01 and ***p<0.001.

Bisphosphonate treatment has been studied extensively in murine models of MM [19–21] and has been implicated to have anti-tumour effects [19]. Here we studied the effect of zoledronic acid treatment in JJN3-bearing mice in a therapeutic approach, given once tumour was established (Fig. 2A); and by a preventative approach, given at the time of tumour cell injection (Fig. 2B). In addition, using a therapeutic approach we assessed the effects of the proteasome inhibitor carfilzomib, which has recently been shown to have bone anabolic effects [17, 22] (Fig. 2B).

Fig. 2A shows representative longitudinal and cross-sectional micro-CT images of tibiae from naïve NSG mice, those injected with JJN3 cells or those injected with JJN3 cells and treated with zoledronic acid from the time of tumour cell injection (Fig. 2Ai-iii). Histological analysis did not show a significant reduction in tumour burden in the tibiae of mice injected with JJN3 cells and treated with zoledronic acid compared to untreated JJN3-injected mice (Fig. 2Aiv). However, zoledronic acid treatment did prevent JJN3-induced trabecular bone loss (Fig. 2Av) and prevented cortical bone lesions (Fig. 2Avi) due to inhibition of osteoclastic bone resorption (Fig. 2Avii). Zoledronic acid treatment had no effect on preventing JJN3-induced reduction of osteoblasts (Fig. 2Aviii).

Fig. 2B shows representative longitudinal and cross-sectional micro-CT images of tibiae from naïve NSG mice, those injected with JJN3 cells, those injected with JJN3 cells and then treated with carfilzomib once tumour was established, or from NSG mice injected with zoledronic acid from the time of tumour cell injection (Fig. 2Bi-iv). Histological analysis showed a significant reduction in tumour burden in the tibiae of mice injected with JJN3 cells and treated with carfilzomib compared to untreated JJN3-injected mice (Fig. 2Bv) whereas, zoledronic acid treatment showed no significant reduction in tumour burden. JJN3-induced trabecular bone loss was not prevented in JJN3 mice treated carfilzomib, only in mice treated with zoledronic acid (Fig. 2Bvi). Similarly, zoledronic acid treatment prevented JJN3-induced formation of cortical bone lesions (Fig. 2Bvii), due to inhibition of osteoclastic bone resorption (Fig. 2Bviii), whereas carfilzomib treatment had no significant effect on lesions or osteoclasts (Fig. 2Bvii & viii). Neither zoledronic acid or carfilzomib treatment had any effect on preventing JJN3-induced reduction of osteoblasts (Fig. 2Bix).

In summary NSG mice injected with JJN3 cells make an ideal short-term model to assess the efficacy of bone modulating drugs used in the treatment of MBD.

### Zoledronic acid treatment prevents U266-induced bone loss and significantly reduces tumour burden, whereas carfilzomib treatment only reduces tumour burden

**Fig 3:**
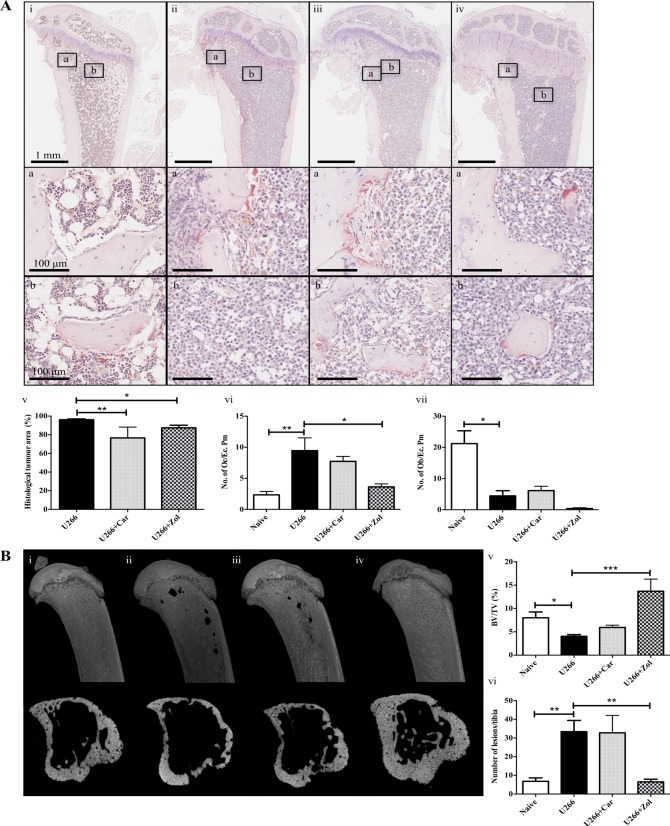
Zoledronic acid treatment prevents U266-induced bone loss and significantly reduces tumour burden, whereas carfilzomib treatment only reduces tumour burden. **A**. Representative longitudinal histological images of tibiae from NSG mice at the end stage of disease, (8 weeks post-injection of tumour cells), injected with vehicle (Naïve) (i), 1x10^6^ U266 cells (U266) (ii), U266 cells treated with carfilzomib (U266+Car) (iii) or zoledronic acid (U266+Zol) (iv). Insets below show the endo-cortical (box a) and trabecular (box b) bone regions. The percentage histological tumour area (v), the number of osteoclasts (No. of Oc./Ec. Pm) (vi) and osteoblasts (No. of Ob./Ec. Pm) (vii) on the cortico-endosteal surface. **B**. Representative longitudinal and cross-sectional micro-CT images at the end stage of disease of tibiae from NSG mice injected with vehicle (Naïve) (i), 1x10^6^ U266 cells (U266) (ii), U266 cells treated with carfilzomib (U266+Car) (iii) or zoledronic acid (U266+Zol) (iv). Micro-CT analysis of trabecular bone volume (BV/TV, %) (v) and the number of cortical bone lesions (vi). Data are presented as mean±SEM and a significant difference from the tumour control group (U266) is indicated, where *p<0.05, **p<0.01 and ***p<0.001.

The effects of zoledronic acid or carfilzomib were assessed in a long-term myeloma model (NSG mice injected with U266 cells). Histological analysis of the tibiae (Fig. 3Ai-iv) showed a significant reduction in tumour burden in mice injected with U266 cells and treated with zoledronic acid from the time of tumour cell injection or carfilzomib once tumour was established (Fig. 3Av).

Zoledronic acid treatment inhibited an increase in U266-induced osteoclastic bone resorption (Fig. 3Avi). However, similar to what was observed in the JJN3 NSG model, zoledronic acid had no effect on preventing U266-induced reduction of osteoblasts (Fig. 3Avii), and interestingly, the number of osteoblasts in this group were lower than the other 2 tumour groups. Carfilzomib treatment was found to have no effect on the numbers of osteoclasts or osteoblasts on the cortico-endosteal bone surface. Micro-CT analysis (Fig. 3Bi-iv) revealed carfilzomib treatment partially, but not significantly, prevented U266-induced trabecular bone loss (Fig. 3Bv) but had no effect on the formation of cortical bone lesions (Fig. 3Bvi), whereas zoledronic acid treatment, as predicted, prevented U266-induced trabecular bone loss and the formation of cortical bone lesions, similar to its effects on JJN3-induced bone disease.

In summary NSG mice injected with U266 cells make an ideal long-term model to assess the efficacy of bone modulating drugs used in the treatment of MBD.

## Discussion

We have shown that JJN3, U266, XG-1, OPM-2 or patient-derived myeloma cells injected into NSG mice result in various levels of BM infiltration. Mice injected with JJN3, U266 or OPM-2 cells had the highest tumour burden with the least variability, whereas XG-1 or patient-derived cells resulted in lower tumour burden which was more variable. The time to onset of disease was also found to vary between the NSG models. This ranged from as little as 3 weeks following injection of the JJN3 cells, to 8 weeks with the other cell lines and patient-derived cells. This data is similar to other reports in which myeloma cells were injected into NSG mice. Variables in other studies included the number of cells injected, the route of administration and the methods of analysis [8, 9, 23].

Tumour infiltration of the BM by JJN3, U266 and OPM-2 cells in NSG mice resulted in significant trabecular bone loss and the formation of cortical bone lesions, whereas bone disease in mice injected with XG-1 cells or patient-derived myeloma cells was more variable. In JJN3, U266 and OPM-2 bearing mice, an increase in cortico-endosteal osteoclast numbers was seen to correlate with an increase in cortical bone lesions.

Our data clearly show that JJN3 cells caused an increase in osteoclastic bone resorption in the tibiae. This is in contrast to Hjorth-Hansen *et al*. [24], who injected JJN3 cells into 4–6 week old female Fox Chase SCID mice and showed a decrease in TRAP positive osteoclasts. They attributed the development of osteolytic lesions to osteoblastopenia, which consequentially reduced bone formation. However, the differences seen between this study and our study may be due to the strain of mice used or the specific anatomical region analysed. In our study, we assessed osteoclast numbers on the cortico-endosteal surface, whereas Hjorth-Hansen *et al* [24] assessed the trabecular bone region. Differences between the cortico-endosteal region and trabecular bone compartments remain interesting but have yet to be fully elucidated.

Miyakawa *et al* [16] conducted histological analysis on the bone disease in sternum and lumbar vertebrae infiltrated with U266 cells. Our results were similar with regards to the presence of osteolytic lesions and increased osteoclast numbers in tumour infiltrated bones. However, they injected 2x10^6^ cells whereas we injected 1x10^6^ U266 cells, which may explain why they saw a more rapid development of disease onset at 6 weeks post-tumour cell injection, compared to 8 weeks in our studies. In addition to histological analysis, we also assessed trabecular bone loss and the development of cortical bone lesions by micro-CT, which clearly showed the full extent of bone disease caused by the presence of U266 cells in the BM.

Fuhler *et al* [13] has investigated the growth of OPM-2 cells in NSG mice when administered by intra-peritoneal injection. They found that a novel SH2 domain-containing inositol-5’-phosphatase 1 selective chemical inhibitor (3α aminocholestane) significantly reduced the growth of these tumour cells and enhanced the overall survival of the mice. However, no bone disease was assessed in this study. Findings from our studies showed that OPM-2 cells, along with U266 cells, provide robust long-term models for investigation of MBD as the tumour infiltration was significant and the variability between mice was low in this setting. In contrast, the injection of XG-1 cells into NSG mice resulted in a less robust model for evaluation of MBD. However, despite the extent of bone disease being very low and the tumour burden being more variable than JJN3, U266 and OPM-2 cell lines, XG-1 cells did infiltrate the BM and grow. To the best of our knowledge, the IL-6 dependent XG-1 myeloma cell line has not previously been injected into NSG mice and this model therefore deserves further evaluation.

As well as the cell lines used in our studies, others have also assessed the use of different myeloma cell lines and patient-derived cells. For example, Chaidos *et al*. [10] demonstrated the growth ability of patient-derived CD138^+^ and CD138 negative (CD138^-^) cell populations administered intravenously into NSG mice, where sorted CD19^-^CD138^+^, CD19^-^CD138^-^ and CD19^+^ cells showed 75%, 25% and 0% BM infiltration respectively. More recently, Schueler *et al*. [11] injected L363, RPMI-8226 and patient-derived myeloma cells into NSG mice. They found BM infiltration by all cells was increased in NSG mice compared to NOD/SCID mice. In addition, they showed that intra-tibial injection of all cells resulted in a higher tumour burden compared to intravenous administration. Others have shown bone disease in NSG mice injected with RPMI-8226 cells, this was prevented when these animals were treated after 3 weeks with oprozomib (an orally bioavailable analogue of carfilzomib) as measured by micro-CT analysis and levels of the bone turnover markers CTX and P1NP in serum [17]. Time to disease onset was also assessed to be 6 weeks post-tumour cell injection.

Interestingly, osteoblast numbers were significantly decreased in all of the NSG myeloma models we assessed, suggesting their effects on osteoblasts were independent from their effects on osteoclasts. This is in agreement with several studies that have shown human myeloma cells secrete several factors that suppress osteoblastic bone formation [25–29].

As predicted, JJN3- and U266-induced bone disease was prevented by zoledronic acid treatment. In the JJN3 model zoledronic acid treatment was given either once tumour was established (a treatment approach) or at the time of tumour cell injection (a preventative approach) and both treatment protocols prevented MBD. However, neither of these treatment approaches showed any anti-tumour effects in mice injected with JJN3 cells. These findings are similar to those observed by Dallas *et al*. observed in the 5TGM1 model [20] and in contrast to what Croucher *et al*. observed in the longer term 5T2MM model [19]. We also chose to investigate the optimal effect of zoledronic acid in the U266 model using a preventative treatment approach. This did result in a significant reduction in tumour burden. These findings may provide some rational for treating patients with monoclonal gammopathy of undetermined significance or smouldering MM i.e. before the onset of MBD with zoledronic acid in order to prevent the onset of MBD, which constitutes symptomatic MM requiring treatment. The mechanism of the apparent anti-tumor effect of zoledronic acid remains controversial. Yet it is tempting to speculate that the prevention of the dysregulation of bone remodeling by zoledronic acid is unconducive to tumor growth. However, the main aim of these studies were to assess the use of bone modulating drugs in the NSG models of MM and zoledronic acid treatment effectively prevented MBD using all treatment approaches. Similar results would therefore be expected using OPM-2 cells, demonstrating that these NSG models are ideal for evaluating bone therapeutic agents. In contrast, NSG mice injected with XG-1 cells or patient-derived myeloma cells may not be as useful due to the low levels of bone disease. Indeed we have injected several primary patient-derived cells into NSG mice and found high variability in tumour burden and bone disease. However, this may not be the case for all patient-derived cells given the heterogeneous nature of the human disease. Others have shown, when patient-derived myeloma cells are administered by intra-tibial injection, greater BM engraftment and reduced tumour variation is achieved [11].

In addition to hypothesising JJN3 and U266-induced MBD would be prevented by zoledronic acid treatment, we investigated the effect of carfilzomib, which has previously been shown to have bone anabolic properties [17, 22]. Despite this, carfilzomib treatment in both the JJN3 and the U266 models only reduced tumour burden and did not significantly prevent myeloma-induced trabecular bone loss or the development of cortical bone lesions. Although in the U266 model carfilzomib treatment partially prevented trabecular bone loss but this was not significant. However, Hurchla *et al* [17], did show oprozomib prevented RPMI-8266-induced trabecular bone loss in NSG mice. Therefore, increasing the dose or duration of carfilzomib may lead to beneficial effects on MBD.

In conclusion, these studies demonstrate that the inoculation of JJN3 cells into NSG mice provides a robust and stable model over a short period of time, with an aggressive disease development as demonstrated by high tumour burden and substantial osteolytic bone disease. The intravenous administration of U266 or OPM-2 cells into NSG mice provide longer-term models, which also feature consistent tumour burden and osteolytic bone disease. It is tempting to propose that the shorter-term model provides a facsimile of aggressive, refractory disease, as typically seen late in the disease course e.g. at relapse. The longer-term models may reflect the less aggressive but nevertheless relentless progression of myeloma in the earlier phases of disease.

In summary, we believe that validation of these short-term and longer-term models provide improved platforms for pre-clinical investigations, tailored to address specific questions relating to the response of tumour burden and MBD to novel therapeutics at various phases in MM.
